# Effect of Local Laser Treatment on the Strengthening of Thin-Walled Structures Fabricated from Non-Alloy Steel

**DOI:** 10.3390/ma16134555

**Published:** 2023-06-23

**Authors:** Oleksandr Kapustynskyi, Nikolaj Višniakov

**Affiliations:** Faculty of Mechanics, Vilnius Gediminas Technical University, Plytines g. 25, LT-10105 Vilnius, Lithuania; o.kapustynskyi@vilniustech.lt

**Keywords:** reinforcing, laser treatment, strengthening, bending, non-alloy steels, thin steel plate

## Abstract

This paper describes the development of new metal-processing technologies that enable the control and improvement of the microstructure and properties of metals. This study investigates the impact of one such technology, laser treatment, on the surface of a thin sheet of non-alloy structural steel. This research aims to address a crucial challenge in expanding the industrial applications of thin-sheet steel products by developing a laser processing technology to create structural strengthening ribs, which can significantly influence the overall strength and stiffness of metal components.

## 1. Introduction

This paper presents an investigation of the effect of local laser processing on the mechanical properties of thin-walled elements or shell structures made of low-carbon non-alloy steels subjected to bending and tensile load. Our previous studies [1,2,3] presented data obtained through analyses of the structure and mechanical properties of non-alloy steel samples with a carbon content of up to 0.3%, including the laser-treated layer. The studies also presented the methodology and results for selecting optimal laser processing conditions, conducting comparative bending and tensile tests on different laser-treated samples, and comparing experimental data with finite element modelling results. The 3D models used in the studies considered the properties of the steel and the laser-treated layer, the sample geometry, and the conditions of bending and tensile tests. The effectiveness of laser processing on the test samples was evaluated using a strength calculation method. A comprehensive analysis of the modelling data, strength calculations, and bending and tensile test results provided insights into the most effective depth of the processed layer and the continuity of the applied tracks. However, certain aspects were not addressed, such as thermodynamic modelling and analyses of phase transformations in steels during laser processing, the selection of processing steps and the directions and placement of the tracks, and the effectiveness of different processing configurations on the final mechanical properties of the treated samples.

The main objective of this research is to evaluate the effectiveness of local laser treatment with surface melting to enhance the bending strength and stiffness of thin-sheet components made of non-alloy steels without the application of complex structural designs or heat treatment. Additionally, the study aims to select the optimal treated layer geometry and local laser processing configuration and parameters, and to modify the local surface areas of thin-sheet non-alloy steel products to increase their operational properties (hardness and bending stiffness).

This study investigates the impact of laser treatment on the microstructure and mechanical properties of bent plates composed of non-alloy steels. The study compares the results of modelling elasto-plastic deformations of various laser-processed samples with those obtained from bending and tensile tests conducted on actual samples. The research findings indicate that localised laser treatment can enhance the bending strength of thin-walled structural elements fabricated from non-alloy steel (1.0402) or similar steels with carbon content below 0.3%.

### 1.1. Overview of Microstructure Formation Features of Non-Alloy Steels

The properties of structural steel during processing are influenced by various structural components, including equilibrium structures, which include austenite and pearlite, as well as non-equilibrium structures like martensite, residual austenite, sorbite, and troostite. The formation of these structures is determined by factors that include temperature, alloy composition, and cooling medium. For example, pearlite has a lamellar structure, while sorbite, troostite, and pearlite differ in their degree of cementite dispersion and hardness [4]. The mechanical properties of the ferrite–cementite structure are affected by the degree of dispersion; finer structures exhibit increased hardness, strength, ductility, and toughness. Among the different structures, sorbite exhibits the highest plasticity characteristics (δ and ψ), while troostite exhibits the lowest.

The transformation of supercooled austenite into bainite occurs within a temperature range below the pearlitic range but above the martensitic range [5,6,7]. When maintained isothermally at temperatures above 350 °C, upper bainite (~HB 450) is formed, characterised by a laminar structure similar to pearlite. In contrast, when maintained isothermally at temperatures below 350 °C, lower bainite (~HB 550) forms, exhibiting a needle-like structure similar to martensite. However, the formation of such a structure requires prolonged isothermal treatment, typically several hours in a furnace, within a temperature range near 350 °C [8].

At extremely high cooling rates exceeding 1200 °C/s, the diffusion decay of austenite is impeded, leading to supercooling and martensitic transformation. Unlike pearlitic transformation, martensitic transformation exhibits polymorphism, which is a non-diffusional or displacive process. The transformation of austenite into martensite is incomplete, resulting in the presence of residual austenite alongside martensite in quenched steel. The wide temperature range of martensitic transformation leads to various types of martensite in steels in the form of plates or laths, which form at different temperatures. Each type of martensite possesses unique properties. The formation temperature of the structural type of martensite is influenced by alloy composition, cooling medium, and other factors. Plate (twin) martensite structure, for example, develops below 200 °C and is a characteristic feature of high-carbon steels (above 0.6% C) and alloy steels [9].

The properties exhibited by the specific martensite type, which appears in the metal structure in the form of plates, are influenced by the presence of a midrib on these plates. This specific martensite type is referred to as twin martensite due to the midrib of each plate being composed of numerous twins. These twins, located on the planes of the martensite plates, have a thickness of 5–30 nm; the hardness of this martensite is 60–75 HRC. Ribbon or packet martensite, on the other hand, is primarily characteristic of the structure of low- and medium-carbon steels, with a carbon content below 0.5% [10]. The temperature threshold above which the martensitic structure is formed in such steels is 300 °C. As its name implies, this martensite type assumes the form of elongated ribbons in one direction, with each ribbon having a thickness of 0.2–2 μm (their length is approximately 5 times greater than their width). The resulting metal structure consists of parallel crystal ribbons, referred to as groups or packets. Between the martensite ribbons, layers of residual austenite can be observed with a thicknesses of 10–20 nm, depending on the alloy type. This particular martensite type exhibits lower levels of hardness (40–60 HRC) but demonstrates increased wear resistance, dynamic viscosity, and ductility compared to plate martensite. Considering the characteristics of the microstructures discussed above, for thin-walled elements made of non-alloy steel with a carbon content below 0.3%, lower bainite or sorbite can be considered to be the preferred options.

### 1.2. Overview of the Features of Processing Non-Alloy Steels

In the metallurgical and metalworking industries, various techniques, such as heat treatment, chemical–thermal processes, and mechanical processing, are employed to manufacture and prepare metals for subsequent processing, as well as to enhance the operational properties of metal products. Among these techniques, heat treatment is the most widely used. When austenite, the high-temperature phase of steel, undergoes cooling, it transforms into different structures depending on the cooling rate; these structures include martensite, bainite, sorbite, and pearlite. Sorbite and bainite are formed through the slow cooling of austenite, while martensite is formed through rapid cooling.

The primary heat treatment methods employed for steels are annealing, quenching, tempering, and normalisation. Although it is not categorised as a fundamental heat treatment, normalisation is frequently employed and is considered a variant of either annealing or quenching, depending on the specific steel grade and workpiece dimensions. The objective of annealing is to attain a steel structure close to equilibrium. To achieve this, carbon steel is subjected to controlled cooling at a rate of 0.05–0.1 °C/s. Annealing leads to a reduction in internal stresses, the enhancement of machinability, the improvement of ductility, and the refinement of the steel microstructure. The cooling rate during annealing is carefully controlled to manipulate the transformation of the steel’s microstructure in accordance with the desired properties [11,12]. Following the annealing process, the hypoeutectoid steel exhibits a microstructure composed of ferrite and pearlite. For low-carbon steels with carbon content of less than 0.3%, normalisation can be employed as an alternative to annealing to achieve a homogeneous fine-grained structure. Normalisation offers advantages in terms of cost-effectiveness due to the reduced cooling time required; the cooling rate during normalisation is typically 1–10 °C/s [13,14]. When steel is cooled from the austenitic state at cooling rates less than the critical cooling rate but higher than the rate necessary for the equilibrium transition of austenite to pearlite, the supercooled austenite undergoes a transformation, resulting in the formation of pearlitic structures [5].

The transformation process during normalisation occurs through diffusion phenomena. When carbon steel is subjected to normalisation with cooling rates exceeding 10 °C/s in an air stream, a dispersed sorbite structure is typically obtained. Alternatively, under faster cooling conditions, such as immersion in oil to achieve a cooling rate of 100–150 °C/s, a troostite structure is formed [11,12].

The formation of bainite in carbon steel is not typically observed during continuous cooling. The intermediate transformation leading to bainite formation can only occur through isothermal holding within a specific temperature range of 500–250 °C, which is typically unattainable in welding or laser processing applications [5]. Achieving the critical cooling rate for quenching, which represents the minimum rate at which all austenite is supercooled and transformed into martensite, is crucial for achieving the desired microstructure. Low-carbon steels with carbon content below 0.3% exhibit the highest critical quenching rate, exceeding 1200 °C/s [15]. Consequently, these steels are often not subjected to conventional heat treatment because typical cooling media are unable to achieve the necessary quenching rate, thus hindering the transformation of austenite into martensite. Carbon steels containing more than 0.3% carbon content are typically subjected to water quenching. For non-alloy steels, the heat treatment method aimed at obtaining lower bainite represents a preferred approach for enhancing the mechanical properties of steel products, despite its inherent challenges. Lower bainite exhibits superior hardness and strength compared to pearlitic structures, while still retaining adequate levels of plasticity and toughness. In contrast, upper bainite is brittle, primarily due to the formation of coarse carbides along the boundaries of ferrite grains. Consequently, upper bainite structures do not significantly improve the hardness and strength of steel.

In addition to the aforementioned heat treatment techniques, various established chemical–thermal surface treatment methods are employed to enhance the operational characteristics of steel components, including cementation, nitrocarburising, and micro-alloying [16,17]. These techniques involve modifying the surface composition and structure of steel components to achieve desired properties such as improved wear resistance, hardness, corrosion resistance, and fatigue strength. The techniques rely on controlled diffusion processes and the interaction of specific elements or compounds with the steel surface to create a modified layer with enhanced properties.

The cementation process is primarily applicable to low-carbon steels with a carbon content of less than 0.2%. However, cementation is often limited to small-sized components with less critical functional requirements. During the cementation process, the austenite phase of the steel transforms into a mixture of ferrite and pearlite beneath the cemented layer. To achieve carburisation of the outer surface layer, the parts are subjected to elevated temperatures of 850–950 °C within a furnace. Steel cementation can be carried out using various media, including solid, liquid, or gaseous carburisers. The cementation process can be time-consuming, with a carbon penetration rate of approximately 0.1 mm/h. Consequently, the resulting thickness of the hardened layer typically does not exceed 0.2–0.5 mm. Therefore, considering the required operational depth of 0.5 mm, the estimated time required to attain the desired strengthening depth is approximately 5 h [18].

Nitrocarburising is a surface treatment method employed for steels, wherein the steel is exposed to a gaseous environment composed of carburising gas and ammonia. This process involves the simultaneous introduction of carbon and nitrogen into the steel structure at elevated temperatures of 700–950 °C. Generally, nitrocarburising is conducted within the temperature range of 850–870 °C. This technique has garnered considerable recognition in the field of mechanical engineering, particularly for components that require the formation of a hardened layer with a thickness not exceeding approximately 0.5 mm to meet operational requirements [19].

Microalloying is a widely recognised and established technique for surface hardening in which minute quantities of alloying elements are introduced into a metal or alloy, with the total mass of these additives not exceeding 0.1% of the original metal or alloy mass. Typical microalloying additives include vanadium, titanium, boron, niobium, zirconium, and various rare earth elements such as cerium, yttrium, and lanthanum, either individually or in combination. Additionally, aluminium, nitrogen, barium, calcium, and magnesium are also commonly utilised. The methodology employed for microalloying is similar to the alloying techniques employed in the metallurgical industry, which aim to enhance the surface properties of the material [20].

Conventional boronising techniques provide consistent outcomes, but face challenges related to low productivity and high resource expenses. Electrolytic and liquid boronising approaches necessitate wastewater treatment, while powder boronising cannot be fully automated and is relatively costly. A notable limitation of these methods is the time required for the process, which can span several hours. To accelerate the boronising process, several alternative approaches have been proposed, including electron-beam, electro-spark, micro-arc, and induction heating methods, as well as plasma spraying. Other techniques involve generating a glowing discharge during powder saturation, utilising ultrasound, employing vibration or pseudo-liquification induced by electrical influence, or employing chemical and physical deposition from the vapour phase [21]. However, despite the reduced saturation duration offered by these methods, their widespread adoption has been limited due to their technical complexity, limited versatility, and high energy consumption. In certain scenarios, a multi-component boronising approach can be employed, in which the component’s surface is saturated not only with boron but also with other elements, including chromium, aluminium, and silicon. The aim of saturation with other elements is to enhance the corrosion and wear resistance of the component’s surface layer. However, the resulting increase in resistance is insufficient for these processes to be widely adopted. After microalloying, the diffusion layer thickness in the presence of alloying elements is typically 20–50 microns, which may be sufficient for components subjected to abrasive wear or corrosive environments [21].

In industrial applications, mechanical surface treatment techniques are employed to induce plastic deformation in thin surface layers, leading to modification of the properties of these layers while preserving the properties of the underlying metal core. This process, known as surface cladding, enhances strength properties, electrical resistance, and the rate of diffusion processes, while reducing the plasticity and corrosion resistance of the treated layer. Surface hardening treatments via plastic deformation are utilised in the final stages of the manufacturing of machine parts. Two types of treatment are commonly employed: dynamic surface plastic deformation (DSPD) and static surface plastic deformation (SPD). Surface cladding can be achieved by bombarding the component with metallic shot, balls, or abrasive particle suspensions; rolling it with rollers, balls, or diamond tools; or stamping. To increase surface hardness through DSPD, rapid surface hardening can be achieved using an explosive substance. Shot blasting is the most frequently employed form of DSPD in a dynamic regime, utilising metallic or corundum shot with particle sizes of 0.5–2.0 mm. The optimal particle velocity during the impact with the treated surface is 50–70 m/s, with a corresponding angle of incidence of 75–90°. The treatment duration is typically limited to 2–3 min, and the cladding layer thickness should not exceed 0.20–0.4 mm. Within the cladding layer, the density of lattice defects increases, altering the grain orientation and shape. As a result, the microhardness of the near-surface layers can be enhanced by up to 50%. For steel, available data indicate an increase in microhardness of 1600–2400 MPa. However, it should be noted that this technique requires relatively high capital investments in a blasting chamber. In addition, this approach is primarily applicable to bulky and thick-walled structures, as thin-walled finished products and components with wall thicknesses below 1.5–2 mm are unsuitable for this method. Also, when exposed to temperatures exceeding 450 °C, all the properties attained through the shot blasting of steel are compromised. Consequently, treated components are unsuitable for subsequent heat treatment. Furthermore, surfaces subjected to cladding lose their corrosion resistance properties [22].

The different heat treatment or thermochemical treatment processes described above and the addition of strengthening elements, complex geometry profiles, and structural thickenings are mostly used to improve the mechanical properties of steel products and their surfaces. But the application of strengthening elements, complex geometry profiles, and structural thickenings increases the manufacturing costs and the weight of metal parts and requires complex equipment and accurate designs. Heat treatment and thermomechanical treatment technologies are expensive, complicated, and long-running processes. These technologies are not appropriate or effective for large constructions from non-alloy and low-carbon steels containing less than 0.3% carbon.

An additional widely recognised and effectively employed technique for localised surface treatment is laser processing, the heating rate of which exceeds 1000 °C/s during laser processing. It should be noted, however, that laser processing encompasses not only rapid heating of the material, but also swift cooling. The cooling of the heated region occurs through heat transfer into the bulk of the metal. Consequently, for more efficient cooling, the total volume of the component should be significantly larger than the volume of the processed zone or be artificially increased via a substantial substrate with considerable thermal conductivity. The cooling rate at temperatures below the melting temperature is typically (5–10)·10^3^ °C/s; during crystallization from the liquid phase, the cooling rate reaches 10^6^ °C/s. This disparity is likely due to the fact that the volume of the welding bath in laser processing is extremely small, and the surrounding metal remains cold [23,24,25].

Hence, laser treatment can also be applied to non-alloy steels containing less than 0.3% carbon. Laser treatment techniques exist both with and without surface melting. In the case of laser transformation hardening using a CO_2_ laser without surface melting, the typical thickness of the hardened layer does not exceed 0.3 mm when employing a laser pulse of 0.15 mm [26]. The effectiveness of laser transformation hardening and the ultimate strength of the laser-treated structure depend on the overall area treated by the laser and the depth of hardening. Consequently, the use of laser transformation hardening without surface melting for reinforcing non-alloy steel structures with less than 0.3% carbon is limited by the hardened layer thickness, as an extensive surface area must be treated. In contrast, laser treatment with a surface melting process yields greater thickness of the treated layer and is preferred for localised laser treatment.

Therefore, laser processing with melting is considered more efficient and advanced in this case, particularly while using the pulsed mode of an Nd:YAG laser, which offers the following advantages: effective processing of thin metals (thickness less than 2 mm); minimal depth of metal heating and the avoidance of overheating; and the ability to adjust the critical power by modifying parameters such as speed, spot size, pulse duration, frequency, and current.

## 2. Materials and Methods

One of the more common types of non-alloy steel for structural purposes (1.0402) with a carbon content of less than 0.3% was utilised in this study (Table 1 and Table 2).

According to the EN 10250-2 standard, the microstructure and mechanical properties of this hypoeutectoid steel may vary depending on the treatment applied. Thin metal plates measuring 20 × 150 × 2 mm were employed, and all samples were tempered to relieve internal stress [27]. After tempering, the microstructure of the steel (1.0402) samples demonstrated a typical hypoeutectoid ferrite–pearlite microstructure, with a hardness of 135 HV. To ensure uniform laser energy absorption, the surfaces of the samples were additionally subjected to blast cleaning using a “Power Plus Tools” (China) sandblaster with “Sakret” (Germany) quartz sand with a grain size of 0.1–0.5 mm. The metal surface roughness, measured as Ra, after sandblasting was less than 5 μm.

## 3. Research Methodology

### 3.1. Laser Surface Treatment

The local laser treatment of metal samples was conducted using the Nd:YAG 4-axis laser-welding machine BMM400 (China) on both sides of the metal samples. The samples were tightly mounted to the work table of the laser machine, and a shielding gas mixture of Ar-CO_2_ (20% CO_2_) was used to protect the surfaces of the samples during laser treatment. Laser treatment of metal surfaces is typically conducted at low energy densities of 10^3^–10^5^ W/cm^2^ [28,29]. The laser processing parameters and laser track geometry were held constant for all experiments (Table 3), with the optimum depth of laser penetration determined in previous research [1,2,3]. The laser processing parameters of the pulsed laser were calculated using a method introduced in previous research and the reference literature [30,31], which considers the physical properties of the steel (Table 2) and the technical characteristics of the 400 W laser equipment used. Based on diagrams presented in prior studies [31,32], the laser processing parameters selected for the steel with an equivalent carbon content (C_eq_) of approximately 0.22% enable processing with melting while attaining a steel hardness of up to 250 HV.

**Figure 1:**
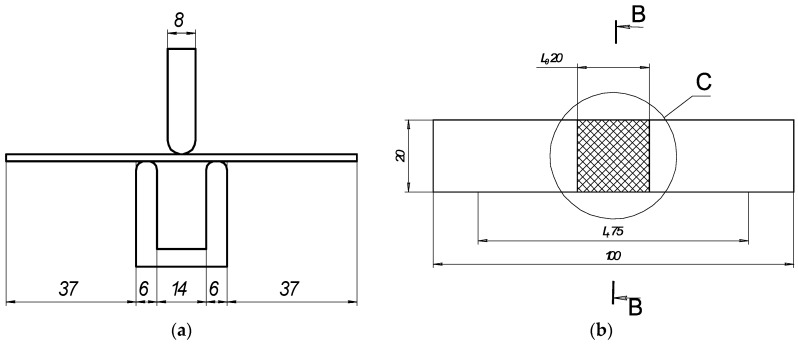
Schematic of bending test: (**a**) general view and sizes of 3-point bending device; (**b**) geometry of samples for bending test, where is C—area of laser processing.

**Figure 2:**
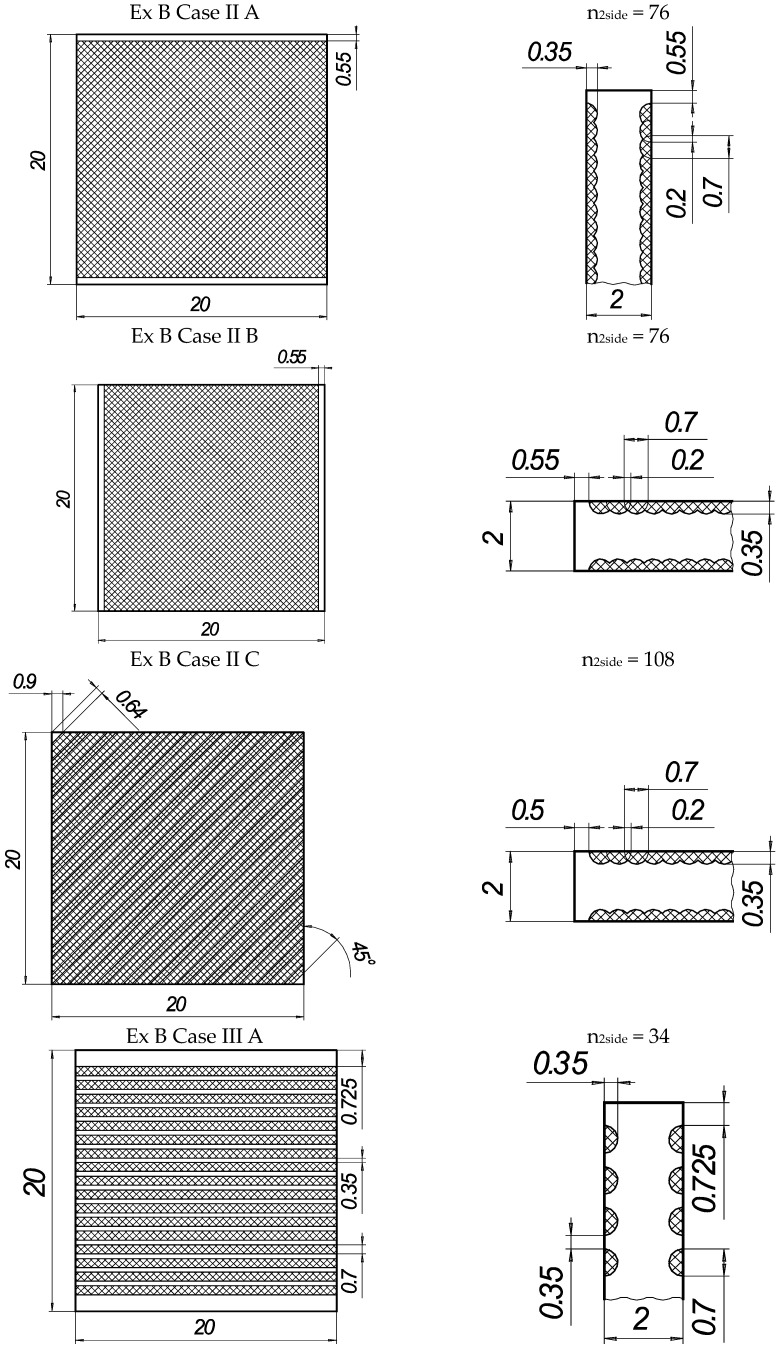

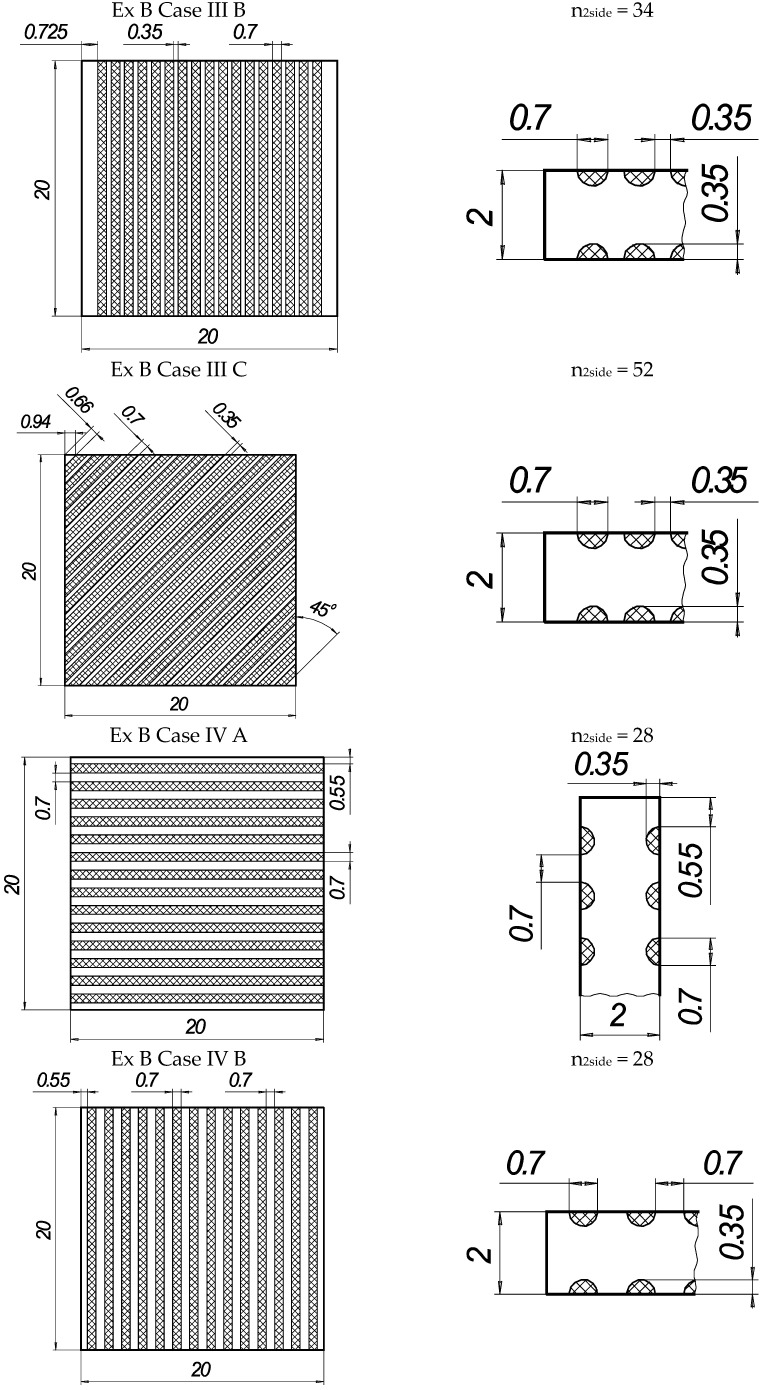

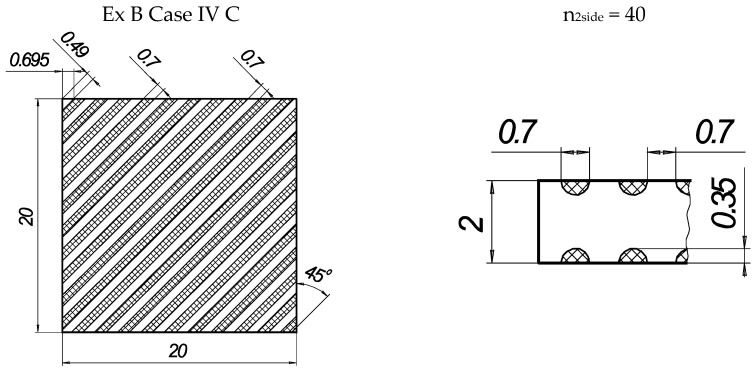
The texture of laser-processed area of samples for three-point bending test.

**Figure 3:**
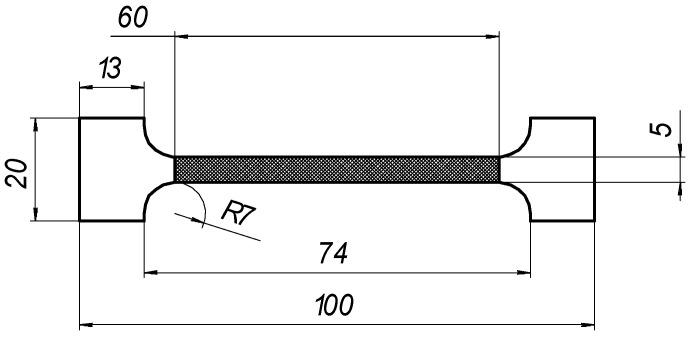
Geometry of samples for tensile testing.

**Figure 4:**
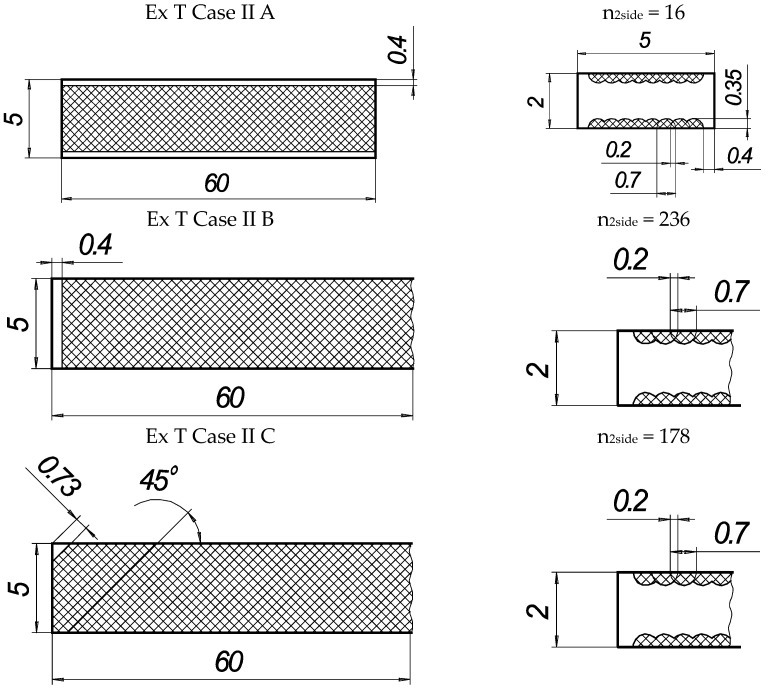

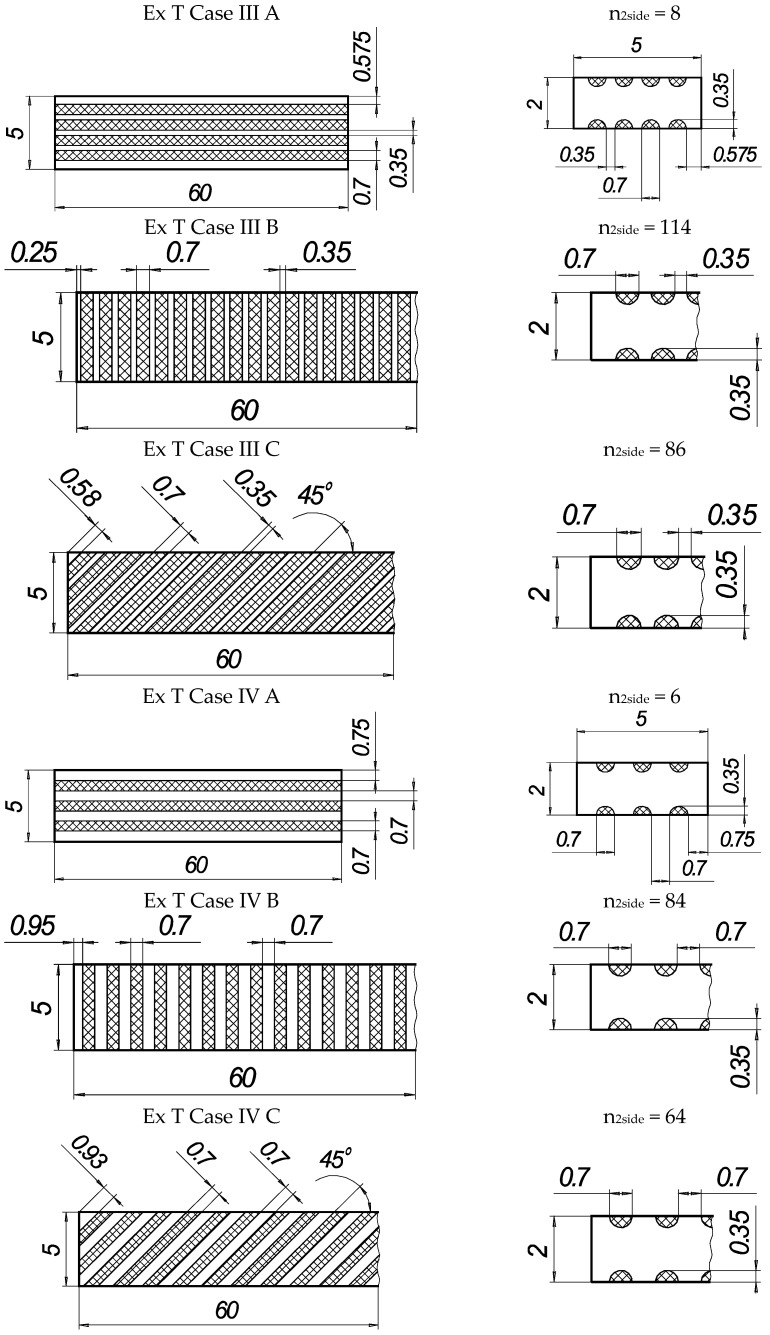
Texture of laser-processed area of samples for tensile test.

In the present experiments, a laser processing depth of approximately 0.35 mm was utilised; such a depth for similar steel samples has been previously discussed in our prior works [1,2,3]. The width of the laser-processed metal track was approximately 0.7 mm. The number of processed sides and the number, orientation, and distance between laser tracks varied. The laser-processed surface area for the bending test, in all cases, was 20 × 20 mm, while for tensile testing, it was 60 × 5 mm. The cross-section of the treated areas of the tensile and bending test samples, the geometry of the laser-processed layers and laser processing cases (with track width and depth, orientation, and distance between tracks) are illustrated in Figure 1, Figure 2, Figure 3 and Figure 4. 

### 3.2. Studying the Kinetics of Phase Transformations in Steels Using Austenite Decomposition Diagrams

In steel processing, the phase and structural transformations that occur at different rates of heating and cooling must be considered. The kinetics and mechanism of austenite transformation during steel cooling can be analysed using isothermal diagrams; however, these diagrams can only approximate the transformations that occur during continuous cooling, as they are based on studies conducted at constant temperatures. Thus, these diagrams are used primarily to develop thermal processing regimes [33,34]. In practice, isothermal transformation is not always achieved, particularly under continuously changing temperature conditions, such as welding or laser processing. During these processes, transformations occur during the continuous cooling of the metal [35,36]. Therefore, in addition to the isothermal diagrams of austenite decomposition (which are necessary for various isothermal treatment methods), thermokinetic (anisothermal) diagrams of austenite transformation are used to provide more accurate assessments of transformations. These diagrams characterise the decomposition of austenite at various cooling rates. The data from these diagrams can provide more precise information on the temperature intervals for phase transformations during continuous cooling and the resulting structural components that are formed from such transformations [37,38]. In complex systems, computational techniques such as CALPHAD (Computer Coupling of Phase Diagrams and Thermochemistry) are utilised to model the behaviour of multicomponent phases. The primary objective of CALPHAD is to advance computational thermodynamics by developing models that represent the thermodynamic properties of various phases, enabling the prediction of properties of multicomponent complex systems and alloys. Various commercial software products (FactSage, MTDATA, PANDAT, MatCalc, JMatPro, and Thermo-Calc) and open-source codes (OpenCalphad, PyCalphad, and ESPEI) [39,40] are publicly available. JMatPro (version 7.0) is a widely used simulation software that computes a broad range of material properties for alloys, with a particular emphasis on multi-component industrial alloys. JMatPro (version 7.0, Surrey Technology Centre, Leicester, UK) [41] was utilised for this study, with the input data listed in Table 2. The analysis of phase transformations was conducted using modules such as TTT/CCT diagrams, as well as phase and properties during the solidification of general steels. This software also simulates approximate stress–strain curves for a particular material at room temperature. However, in this study, experimental data were used to ensure the reliability of the FEA simulation results.

### 3.3. Experimental Methods of Investigation of the Materials’ Properties and Microstructure

The mechanical properties of the base (laser unprocessed) metal, including the stress–strain diagram, modulus of elasticity E_b_, offset yield stress (proof stress) σ_0.2,B_, ultimate and fracture stresses, as well as the fracture strains ϵ_(ul,b)_, were determined via a standard tensile test according to LST EN ISO 6892-1 [42]. The tensile testing results are presented in previous studies [1,2,3].

The corresponding mechanical properties of the laser-processed metal, i.e., E_l_, σ_0.2,l_, and σ_0.2,B_, were obtained indirectly using the empirical relationships between the corresponding mechanical properties and hardness, which was determined according to EN ISO 6507-1 [43]. A series of three samples were used for the mechanical tests; the estimated means $m_{x}=\sum_{i=1}^{N}x_{i}$ are presented hereafter and were used for the calculations presented below. Machined samples of type B, with initial gauge lengths of L_0_ = 50 mm and L_c_ = 60 mm, and an initial cross-section of S_0_ = 10 mm^2^, were used for the tensile test.

Elastoplastic bending of the thin plate was conducted using a 3-point bending instrument (Figure 1a) according to LST EN ISO 7438 [44]. The radius of the mandrel was specified for normal-strength steels according to the dependence D/2 = 2a. The distance between the supports was determined using the standard equation l = D + 3a.

The resulting distance between supports was 14 mm, and the widths of the mandrels were each 8 mm (Table 4). The test was conducted using the strain control mode with a rate of displacement of 1 mm/s. The load F_exp_ ranged from 0 N up to the maximum load of F_exp_ < 2000 N. The deflections were measured at the middle of the span of the plate.

The geometries of the machined test pieces for tensile testing conformed to the specifications provided in Annex B of LST ISO 6892-1 (Table 5). The testing rate based on strain rate control (method A) was used.

A universal tensile testing instrument, 2055 P-5 (Tochpribor, Ivanovo, Russia), with a bending test tool, Labview version 2020 (National Instruments, Austin, TX, USA) software, and PXI system hardware (chassis NI PXIe-1073, Austin, TX, USA and controllerNI PXIe-4330, Austin, TX, USA) were used for the tensile and bend tests. A 5 kN tension dynamometer was used for the tensile test, while a 2 kN compression dynamometer was used for the bending test.

Steel hardness on the surface with a load of 10 N was determined using a ZHU universal hardness tester (Zwick/Roell, Swtzerland) following the Vickers method. The hardness of the laser-processed layer was determined using a ZHμ hardness tester (Zwick/Roell, Switzerland) with a diamond square-based tetrahedral pyramid tip with a load of 2.942 N. The hardness was measured on the surface of the laser-processed layers and on the sample’s cross section.

The chemical composition of the steel was determined using a PMI Master PRO (Oxford Instruments, Abingdon, UK) optical emission spectrometer.

Metallographic examination of the steel and the laser-processed layer, analysis of the geometry, and determinations of the dimensions of the laser-processed layer were performed using an Eclipse MA200 optical microscope (Nikon, Tokyo, Japan) with a Lumenera Infinity 2-2 video camera and a JEOL JSM-7600 (Tokyo, Japan) scanning microscope with an energy-dispersive spectrometer (EDS) (INCA Energy X-Max20, Oxford Instruments, Abingdon, UK) at different magnifications (up to ×1500).

The microscopic analysis technique using reagents for colour etching [45] served as an additional tool for examining the phase composition. In this study, several of the most popular sets of colour metallographic etchants were used to identify various possible structural constituents in steels, as described below.

- “Standard” acid etchant complex based on a combination of picric and nital [46]: Reagent 1—2.4% solution of picric acid in ethanol; Reagent 2—1.4% solution of nitric acid in ethanol. The sample was sequentially etched for 2 s in Reagent 1 and up to 10 s in Reagent 2. Picric is considered to be the best acid etchant for revealing the structure of ferrite, pearlite, or dispersed varieties of pearlite and bainite in low-carbon steels. Etching steels with this method colours the structures of sorbite and troostite in brown or tan, martensite in blue, and austenite in a pale orange.
- “Tint” colour etchant based on sodium metabisulfite was used [47]: Reagent 1—4% solution of picric acid in ethanol (4 g dry picric acid/100 mL ethanol); Reagent 2—aqueous solution of sodium bisulfite (1 g Na_2_S_2_O_5_/100 mL distilled water). The sample surface was pre-etched with Reagent 1 (4% picric) for 15 s. After pre-etching, the specimen was etched with Reagent 2 for 15 s. The sample was oscillated during the entire pre-etching and etching stages. After each stage, the sample surface was washed with water and ethanol and dried in warm air. Aqueous sodium metabisulfite is an effective colour etchant for mixed microstructures because it reveals ferrite grain boundaries, pearlite or dispersed varieties of pearlite, bainite, and martensite. Etching steel samples using this method colours the structures of sorbite and troostite in brown or tan, bainite in blue, martensite in light brown, ferrite in white, and austenite in tin white.
- “Tint” colour etchant based on sodium thiosulfate [48]:
  Reagent 1—4% solution of picric acid in ethanol (4 g dry picric acid/100 mL ethanol); Reagent 2—10 g Na_2_S_2_O_3_ and 3 g K_2_S_2_O_5_/100 mL distilled water.
  The sample surface was pre-etched with Reagent 1 for 15 s. After pre-etching, the specimen was etched with Reagent 2 for 30 s under constant oscillation. This method of etching steels colours the structures of lower bainite in blue and grey, martensite in light brown, and cementite in white.

Qualitative X-ray diffraction (XRD) analysis of the phase composition of materials was performed using a SmartLab diffractometer (Rigaku, Tokyo, Japan) with a 9 kW rotating anode X-ray source, Johansson Kα_1_ optics, a CALSA ultra-high resolution spiral analyser, and the PDF4 database.

Mössbauer spectroscopy was carried out using a Mössbauer spectrometer (Wissenschaftliche elektronik GMBH, Starnberg, Germany) to investigate the physical properties and characterise the phase composition of the layer treated via laser [49].

The measurements were performed in both transmission geometry and conversion electron geometry. Magnetic pure iron spectrum splitting was used for velocity calibration. A clean iron foil (thickness 25 μm) was used as a velocity scale calibrator when measuring the standard sample. Samples up to 10 × 10 mm^2^ in size were used. The WinNormos software package was used for mathematical descriptions of the spectra. The concentrations of identified substances in the test samples were determined by comparing the area under the sample spectrum with the area under the spectrum of the standard (α-Fe foil).

### 3.4. Simulation of Thermal Cycles during Laser Welding

Since the object of processing was thin steel plates rather than complex-shaped metal structures, relatively simple computational methods based on analytical solutions to the conduction heat flow equation [50] were used to calculate and analyse temperature fields during the laser processing of steel. Modelling of the shape and depth of the melted metal volume during laser processing of the steel plates was performed using the software package Smartweld version 3 [51,52]. This software provides interactive displays of the dimensions of the fusion zone and heat-affected zones, as well as temperature contours as a function of laser parameters, by solving conduction heat transfer models. An add-on program, ISO-3D GAUSS, was utilised to determine the temperatures adjacent to the weld. This program is valuable for simulating defocused laser beams and other distributed heat sources, allowing for the specification of a spot diameter up to 5 mm wide. The model is based on the Eagar and Tsai integral solution to the conduction heat flow equation for a moving Gaussian distributed source. Typical thermal properties for plain carbon steels were used in the calculations, including a thermal conductivity of 51 W/mK and specific heat capacity of 462 J/kg-K. The laser welding parameters used in this study included a spot diameter of 3 mm, a welding speed of 240 mm/min, a base ambient temperature of 20 °C, and an average laser power of 150 W.

### 3.5. FEA Simulation of Tension and Bending of Thin Metal Plate

**Figure 5:**
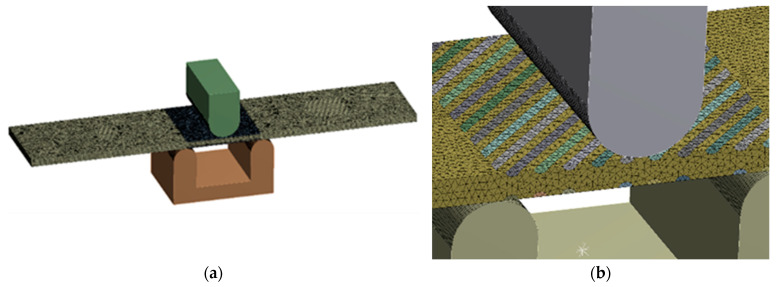
Numerical model for bending simulation: (**a**) numerical mesh; (**b**) enlarged view of laser track mesh.

**Figure 6:**
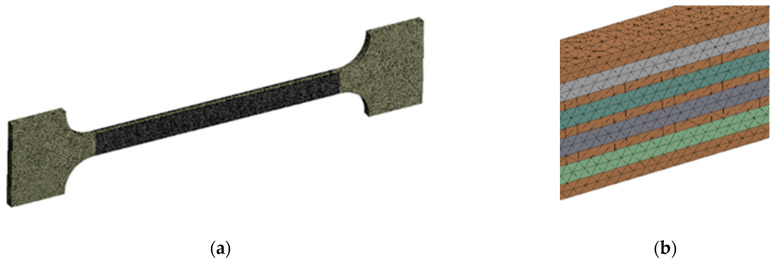
Numerical model for tensile testing simulation: (**a**) numerical mesh; (**b**) enlarged view of laser track mesh.

The tension and bending of the laser-treated thin metal samples were simulated via finite element analysis (FEA). The Ansys workbench software package (version 16.0) was used for mechanical stress–strain simulation and analysis [53,54]. Numerical models of the bending and tensile tests were created that replicated the experimental test devices. General views of the numerical models are shown in Figure 5a and Figure 6a. The geometry and dimensions of the FEA models of the samples were identical to those of the samples used in the experimental investigation (Figure 2 and Figure 4).

The distance between supports (14 mm), mandrel diameter (8 mm), and fixation conditions in the bending FEA simulation were the same as those used during the real bending experiments. The plates in the FEA modelling were fixed, as shown in Figure 5. For all variants, the load was applied in the middle of the span of the stand (Figure 5a). The laser processing cases of the bending samples used in the FEA are summarised in Figure 2.

Samples of the same geometry and sizes were used in the FEA simulation of tensile testing, including the same original gauge length (50 mm) and free length between grips (74 mm). The tensile samples in the FEA modelling were fixed, as shown in Figure 6. The laser processing conditions of the tensile samples used in the FEA are summarised in Figure 4.

3D solid brick and tetrahedral elements were used to discretise the complex geometry of the modelled samples with laser-processed layers [55]. Large-scale finite elements up to 0.7 mm were used to mesh the laser-unprocessed parts of the sample, while a finer mesh, with finite element sizes up to 0.12 mm, was adopted for the discretisation of the laser-processed layer. The differences between the mechanical properties of the base metal and the laser-processed layer used to simulate elastoplastic deformation of the tensile and bending samples are presented in Table 6.

The properties of the steel and the deposited layer used in the calculations were determined both experimentally and predicted using JMatPro software (version 7.0), considering the exact chemical composition of the layers. JMatPro software (version 7.0) can predict various alloy properties over a wide range of temperatures. However, in our calculations, only mechanical properties at room temperature were considered, which were obtained through experimental mechanical tests of the samples. For discrepancies between the values obtained through JMatPro and the experimental mechanical properties during the modelling process, real values of the processed layer and steel properties were used for greater accuracy, although discrepancies between the experimental and calculated results did not exceed 5.5%. The yield strength and shear modulus values differed by less than 4%, and the ultimate tensile strength values differed by 2.5–5.5%. There was no observed difference the experimental and calculated elastic moduli and Poisson’s ratios. Therefore, the method of data collection for material properties in this study did not have a significant impact on the final modelling results.

## 4. Research Results

### 4.1. Results of the Structural Analysis of the Laser-Processed Surface Layer

**Figure 7:**
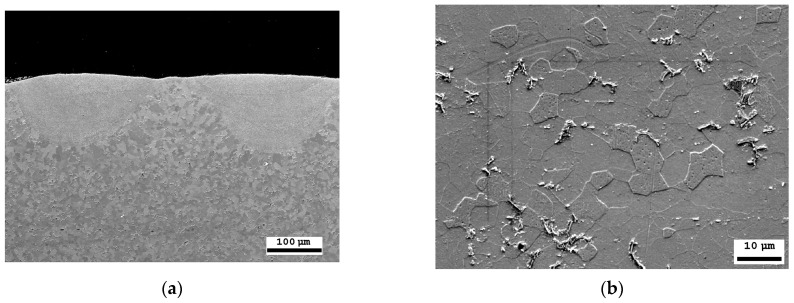
SEM microscopic examination: (**a**) cross-sectional view of the laser-treated area; (**b**) laser-untreated area of steel with a ferrite–pearlite structure.

The thickness of the laser-processed layer, determined via metallographic examination of cross-sections of samples of laser-processed metal, is about 0.35 mm (Figure 7a). The results indicate that there are no unacceptable inclusions, porosity, or internal defects in the remelted area or transition area. The microstructure of the base metal consists of 70% ferrite and 30% pearlite (Figure 7b). The granularity in the processed zone decreases from G8 (average grain diameter—18 µm) to G10 (average grain diameter—10 µm), according to ISO 643 specifications [56].

The diffraction peaks of α-Fe dominate in the XRD patterns of the base metal and the treated area. The low-intensity diffraction peaks are attributed to SiC refractories and iron oxides (FeO, Fe_3_O_4_). These component traces remained on the analysed surface after the surface polishing procedure.

**Figure 8:**
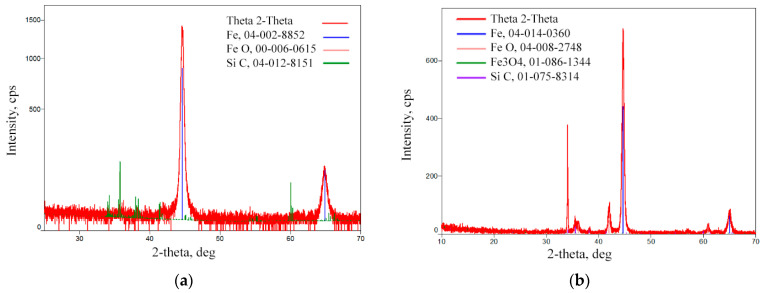
XRD pattern of the laser-processed layer: (**a**) surface of basic metal; (**b**) area of treated layer.

According to the XRD data analysis (Figure 8), the X-ray diffraction pattern of the laser-processed layer is typical of the ferrite microstructure family (including sorbite and troostite), with a BCC crystal structure. Martensite and bainite have this same crystal structure. An Fe_3_C peak is not observed as expected, indicating that the carbon content in the steel is low or that the peak sensitivity is below the sensitivity detection limit. Therefore, the XRD analysis confirms that there is no unstable austenite retained in the laser-processed area, because the austenite has an FCC crystal structure and other XRD diffraction peaks.

**Figure 9:**
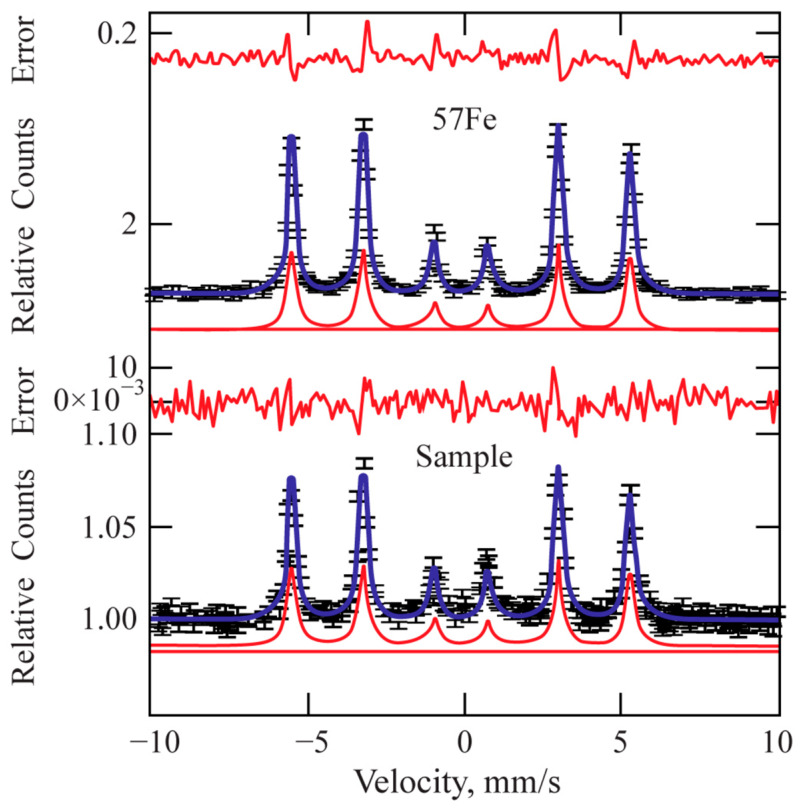
Mössbauer pattern of the laser-processed layer.

Mössbauer spectroscopy applied to the treated surfaces shows that the main phase is Fe. Figure 9 shows a comparison with the spectrum of standard isotropic iron (57Fe). The total concentration of the other phases (carbides) in the processed sample is within the margin of the measurement error (2%). Therefore, Mössbauer spectroscopy only confirms the absence of any unstable phase within the laser-treated area.

In non-alloy steels, high levels of retained austenite are typically found, together with martensite or bainite phases, in the quenched steel after cooling. The microhardness measurement shows that the hardness of the laser-processed layer increases up to 200 HV (by 60%) compared to the laser-unprocessed base metal hardness. Based on the hardness measurement results, there are no hard and brittle bainite and martensite microstructures in the laser-processed areas, or their concentration is very low, because such quenching microstructures have the highest hardness values: bainite—400 HV, martensite—450 HV [34,57]. Consequently, to achieve a hardness of approximately 200 HV, it is necessary to have a structure with a minimum of approximately 25% bainite or martensite in the present non-alloy steel after rapid cooling of the microstructure of the melted area, or the microstructure must be strengthened by refining the grain size and creating a finely dispersed perlite (e.g., sorbite, troostite) structure.

The microstructure located in the laser-processed area of the non-alloy steel sample exhibits a typical sorbite structure. The distance between the lamellae, measured via SEM, is around 0.3–0.5 µm. This distance is typical for sorbite, because perlite has a distance between the lamellae of 0.6–0.7 µm, troostite—0.1 µm, and martensite and bainite—0.2 µm, and the thickness of the retained austenite lamellae is 0.05–0.2 µm [10]. The hardness of the laser-processed layer also corresponds to the typical hardness of sorbite, which is 200–300 HV [58].

**Figure 10:**
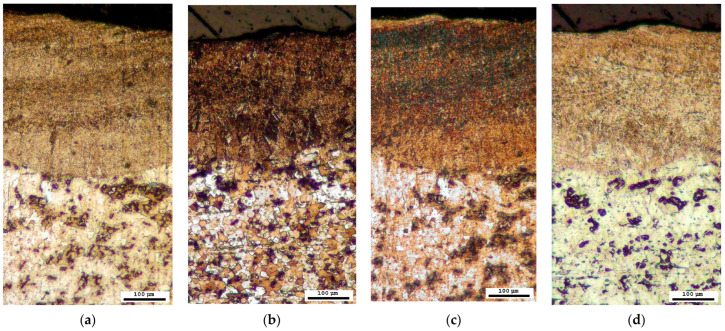
Microstructure after colour treatment (optical microscope): (**a**) solution A; (**b**) solution B; (**c**) solution C; (**d**) solution D.

The phase composition was further investigated through microscopic analysis by employing four distinct reagents for colour etching. Each of the methods described in Section 3.3 confirmed the presence of a sorbite structure, as evidenced by brown or dark brown colouration, and the absence of a harder bainite structure, which would have exhibited blue or grey colouration, in the region affected by the laser (Figure 10).

**Figure 11:**
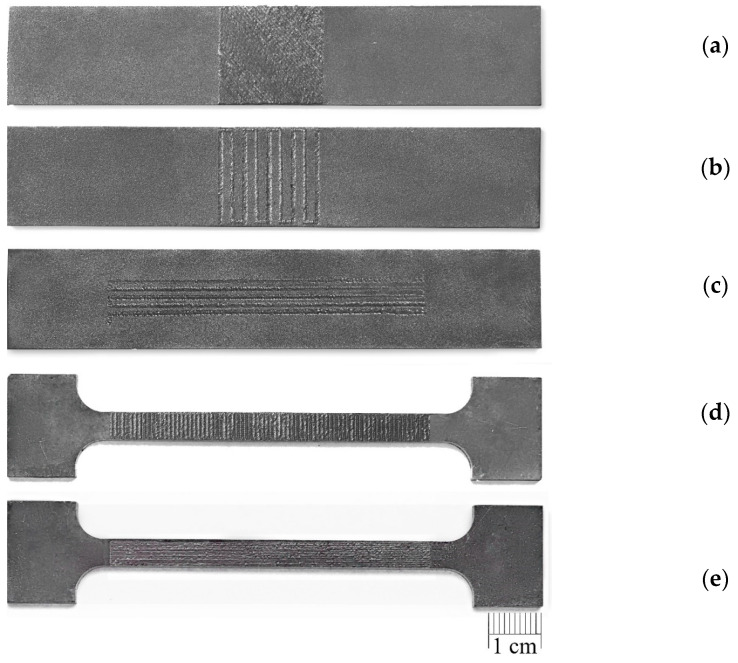
General view of laser-treated plates: (**a**) Ex B Case II C; (**b**) Ex B Case IV B; (**c**) Ex T Case III A; (**d**) Ex T Case II B; (**e**) Ex T Case IV C.

The sorbite structure of the laser-processed layer was formed due to the applied high overlap coefficient of laser spots, with associated additional heating and partial remelting of the crystallised pool during the next laser pulse. This effect allowed the cooling rate of the melted pool to be reduced and prevented the formation of more brittle quenched structures in the laser-processed layer. A general view of the laser-treated plates is illustrated in Figure 11.

The sorbite structure is advantageous because it has a finer texture, with higher dispersity and stiffness than pearlite, which increases the mechanical strength and wear resistance of the laser-processed metal parts. It also does not exhibit any loss in plasticity, which is typical for hard and brittle quenching (martensite or bainite) microstructures [59].

### 4.2. Results of Analysis of Phase Transformation Kinetics in Steels Using Austenite Decomposition Diagrams

**Figure 12:**
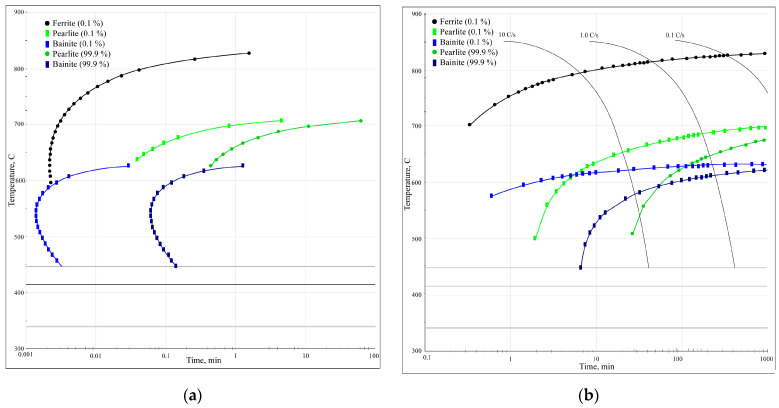
Phase transformation kinetics analysis diagrams: (**a**) TTT diagram; (**b**) CCT diagram.

The phase transformation kinetics analysis diagrams (Figure 12) illustrate that for this particular steel, at low cooling rates below 10 °C/s, only diffusional austenite decomposition occurs, yielding a ferrite–cementite structure with varying degrees of dispersion (i.e., pearlite, sorbite, and troostite). Increasing the cooling rate above 10 °C/s suppresses pearlitic transformation and results in austenite decomposition to form bainite. This intermediate transformation region is characterised by incomplete transformation, resulting in the presence of other phases after cooling (including residual austenite). Based on the results of the diagram analysis, the most optimal cooling methods for laser-treated surfaces are air cooling or a compressed air (or protective gas) jet, which both provide the necessary cooling rate (around 10 °C/s) for the formation of a sorbite structure.

### 4.3. Results of Heat Transfer Modelling during Laser Treatment

**Figure 13:**
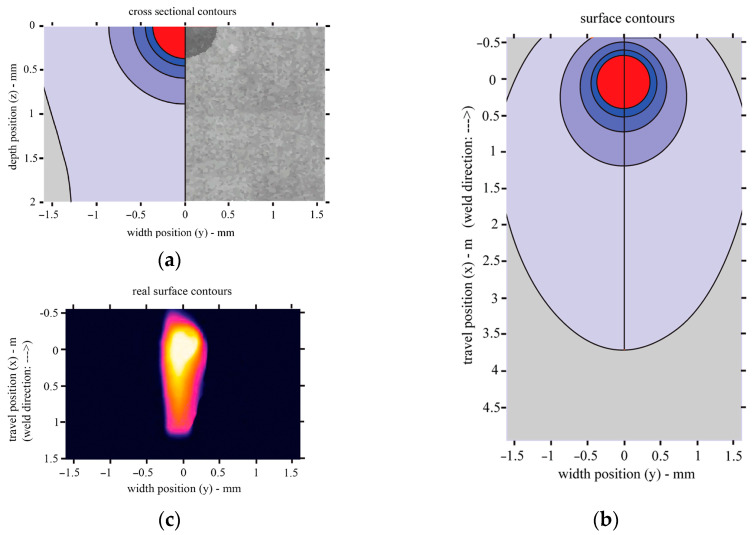
Heat transfer during laser welding: (**a**) cross-sectional view of the model and a comparison of the model and melted pool observed experimentally; (**b**) top view of the model; (**c**) top view from the Optris Infrared Camera PI450i.

By modelling the heat distribution during laser treatment, it was found that the shape of the molten pool and the achieved depth of melting are very close to the values obtained experimentally. For the values of the laser treatment parameters used in the model, the depth of melting reaches 0.35 mm and the width is approximately 0.7 mm. In addition, the width of the heat-affected zone (in the brittle zone, where the temperature is above 320 °C) under such laser treatment conditions is 3.4 mm (Figure 13).

### 4.4. Results of the FEA Simulations of the Bending and Tension of the Laser-Processed Samples

**Figure 14:**
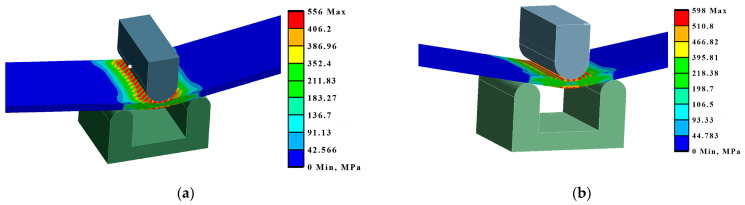
Maximum deformations and equivalent von Mises stresses in the samples: (**a**) equivalent stresses in Ex B Case III C; (**b**) deformations in Ex B Case IV B.

By modelling the processes of stretching and bending of the laser-treated samples using FEA, the required data were obtained for comparative analysis and determination of the efficiency of the laser treatment. The simulation results were compared with the experimental results, i.e., mechanical testing of samples. According to the modelling results, under the bending conditions, according to LST EN ISO 7438 [44], the most effective treatment method out of the nine options that were tested is the processing of the surface with roller overlap (Ex B Cases IV A, B, and C). From the modelling results, the load required for the same bending (to the same bending angle value of ~120° and plate deflection of ~27 mm) of plates with different laser treatments is strongly dependent on the volume of the remelted layer, and the most effective method is remelting with roller overlap. In this case, the orientation of the treatment (rollers) does not have any significance. The increase in the load required for the same deflection of laser-treated samples in Ex B Cases IV A, B, and C, compared to samples with an untreated laser surface, is 25–27% (Figure 14, Table 7).

**Figure 15:**
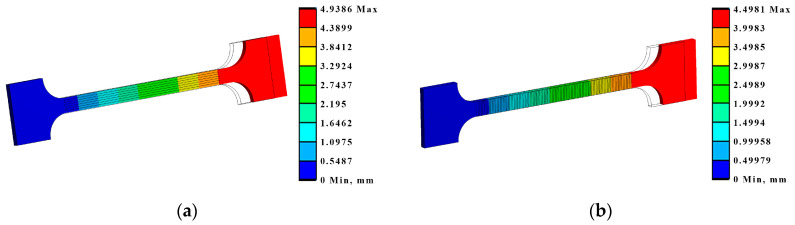
Maximum deformations and equivalent von Mises stresses in the samples: (**a**) deformations in Ex T Case IV A; (**b**) deformations in Ex T Case III B.

Also, based on the modelling results, under the tensile conditions, according to LST EN ISO 7438 [44], the most effective treatment method is the processing of the surface with roller overlap (Ex T Cases IV A, B, and C). In these cases, the relative elongation of the samples in the measurement zone decreases by 33–38% compared to the untreated sample (Table 8, Figure 15).

### 4.5. Results of the Mechanical Testing of the Laser-Processed Samples

**Figure 16:**
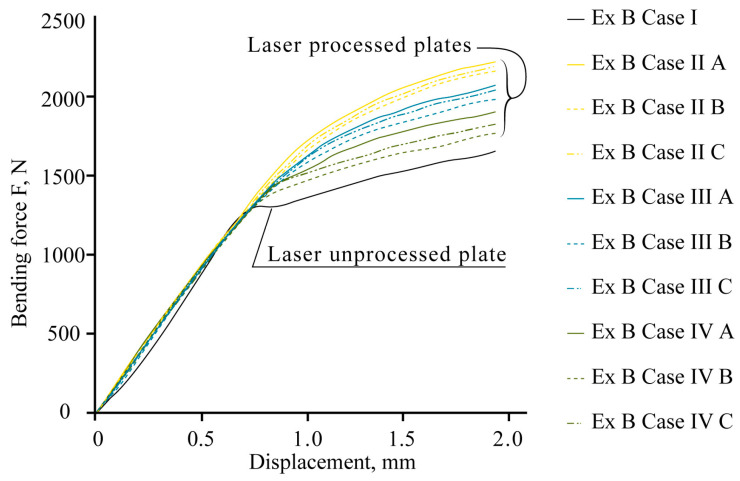
Results of bending tests.

The mechanical bend tests show that local laser processing increases the load required to reach the same level of deflection of laser-processed samples compared to those that are unprocessed (Figure 16).

The experimental data confirm the effect of strengthening, and the total resistance of the samples to bending is influenced by the position of the laser-processed area, the distance between laser tracks, the volume of the hardened phase, and its ratio to the volume of laser-untreated material in the area of maximum stress from the applied load.

The maximum bending load required to reach the deflection limit in the elastic stage varies, depending on the applied variant of laser processing. For example, the experimental bending load, F_exp_, increases depending on the laser-processed case: for 0.5 mm deflection, F_exp_ increases from 3 to 27%; for 1.0 mm deflection, F_exp_ increases from 29 to 48%; for 1.5 mm deflection, F_exp_ increases from 32 to 55%; and for 2.0 mm deflection, F_exp_ increases from 30 to 52%.

**Figure 17:**
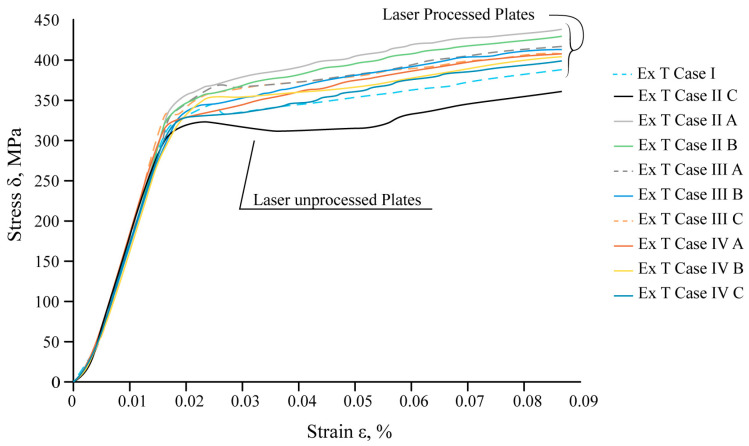
Results of the tensile tests.

The mechanical tensile tests show that local laser processing increases the load required for the destruction of laser-treated samples compared to untreated ones. The results of the mechanical tensile tests of steel samples are summarised in Figure 17.

The experimental data confirm that the total resistance of the samples to destruction is influenced by the position of the laser-treated area, the distance between laser tracks, the volume of the hardened phase, and its ratio to the volume of laser-untreated material.

The maximum tensile load required to achieve destruction of the sample varies depending on the applied variant of laser processing. For 45° reinforcing ribs with an overlap (Ex T Case II C), an additional 9% load should be applied compared to the overlapping vertical ribs (Ex T Case II B), and an additional 5% load should be applied compared to the untreated sheet (Ex T Case I).

### 4.6. Comparison of the Experimental and Modelling Results

The FEA model was validated by comparing the bending and tensile test results with the results of the experiments. The experimental results and deflection simulation results of the laser-untreated samples were compared with the results of the samples treated, applying different distances between laser tracks, different position of laser tracks, and different lengths between the supports for the bending test. Based on the experimental and modelling results, the degree of improvement depends on parameters such as the distance between laser tracks and their orientation. The track orientation should be chosen based on the expected loads on the sample, as different orientations are preferable for bending and stretching. However, processing with overlap remains the most effective method of treatment.

Detailed comparisons of results of the mechanical bend tests and computational modelling are summarised in Table 9. Based on the results of the bending tests in Table 9, the most effective variant for processing thin-walled plates is found to be double-sided processing with overlapping (Ex B Case II A). It is observed that achieving a deflection of 2 mm in a laser-treated sample requires 33% more force compared to a laser-untreated sample.

The experimental results and tensile simulation results of the laser-untreated samples were compared with the results of samples treated, applying the maximum force to break the specimens in real experiments and the maximal applied force of 3750 N in FEA modelling. A comparison of all the obtained results for the mechanical tensile tests and computational modelling is summarised in Table 10. The values of the maximum equivalent stresses can be determined as preliminary and cannot be predicted very accurately due to the large error (more than 20%) between the experiment and the modelling values. An error could have been introduced by existing laser processing microdefects or the sample structure, or other microdefects obtained after preparing the samples for tensile tests. The most significant increase in the required tensile load and available stresses, according to the modelling results, is in the double-sided laser-treated samples with ribs positioned at 45° and with an overlay of 0.35 mm (Ex T Case III C) (equivalent stress is 26% higher at 558.24 MPa compared to the laser-untreated sheet at 410.57 MPa). Based on the obtained results, it can be concluded that increasing the distance between the tracks from overlap to 0.7 mm significantly reduces the maximum processing efficiency.

## 5. Conclusions

Our experimental results and computer simulations of elastoplastic deformation established that local laser processing with surface melting can be used to strengthen the structural elements made from thin-sheet 1.0402 (EN 10250-2:2022) non-alloy steel with a carbon content of up to 0.3%, rather than the application of complex geometric shapes, additional strengthening elements, or heat treatment. In this case, the stiffness and strength of metal sheet parts were improved due to local changes in the microstructure and properties of the steel. A thin sorbite structure was formed in the melted area, which increased the hardness of the steel samples in the treated areas compared to the areas without laser treatment, based on 1.0402 (EN 10250-2:2022), from 149 HV to 200 HV.

This study establishes that the stiffness and mechanical strength of laser-treated thin-sheet steel are influenced by the laser parameters (the area and texture of the laser processing). The difference in spacing between laser tracks and their orientation affect the degree of mechanical strength increase in thin-sheet steel.

## Figures and Tables

**Table 1:** Chemical composition of samples from steel (1.0402).

Chemical Elements (wt%)								
C	Si	Mn	P	S	Cr	Mo	Ni	Fe
0.18	0.18	0.45	0.02	0.02	0.05	0.01	0.07	rest

**Table 2:** Mechanical properties of samples from steel (1.0402).

Elastic Modulus E (GPa)	Yield Strength σ_0.2_ (MPa)	Tensile Strength σ_B_ (MPa)	Relative Extension A (%)	Hardness (HV)
210	256	410	30	135

**Table 3:** Bending (B) and tensile (T) testing sample geometry.

Experiment Name	Number of Ribs (Total)	Overlay Coefficient of Laser Processing on the Sheet	Position and Stress–Strain State of the Laser-Treated Layer	Position of the Ribs
Ex B Case I	0			
Ex B Case II A	76	Overlap	Double-sided	Horizontal
Ex B Case II B	76			Vertical
Ex B Case II C	108			45 degrees
Ex B Case III A	34	0.35		Horizontal
Ex B Case III B	34			Vertical
Ex B Case III C	52			45 degrees
Ex B Case IV A	28	0.7		Horizontal
Ex B Case IV B	28			Vertical
Ex B Case IV C	40			45 degrees
Ex T Case I	0			
Ex T Case II A	16	Overlap	Double-sided	Horizontal
Ex T Case II B	236			Vertical
Ex T Case II C	178			45 degrees
Ex T Case III A	8	0.35		Horizontal
Ex T Case III B	114			Vertical
Ex T Case III C	86			45 degrees
Ex T Case IV A	6	0.7		Horizontal
Ex T Case IV B	84			Vertical
Ex T Case IV C	64			45 degrees

**Table 4:** Bending test parameters and geometry of the samples.

Thickness of the Test Piece a (mm)	Width of the Test Piece b (mm)	Length of the Test Piece L (mm)	Distance between Supports l (mm)	Diameter of Mandrel D (mm)	Radius of Supports R (mm)	Rate of Displacement v (mm/s)
2	20	100	14	8	3	1

**Table 5:** Tensile testing parameters and geometry of the samples.

Original Width of Parallel Part b_0_ (mm)	Original Thickness a_0_ (mm)	Original Gauge Length L_0_ (mm)	Parallel Length L_c_ (mm)	Free Length between Grips L_f_ (mm)	Total Length of Test Piece L_t_ (mm)	Length of Gripped Ends C (mm)	Transition Radius r (mm)	Width of Gripped Ends B (mm)	Strain Rate e_Le_, S
5	2	50	60	74	100	13	25	20	0.002

**Table 6:** Mechanical properties of steel samples.

Material	Modulus of Elasticity, E (GPa)	Shear Modulus, G (GPa)	Yield Strength, σ_0.2_ (MPa)	Ultimate Strength, σ_B_ (MPa)	Poisson’s Ratio ν	Strength Coefficient E_1_ (GPa)
Base metal	200	78.1	256	410	0.28	0.512
Laser-processed layer	210	82	412	662	0.28	0.843

**Table 7:** Bending modelling results; maximum equivalent stresses and deformations of samples.

Imposed Vertical Displacement of the Point (mm)	0.5	1.0	1.5	2.0
Laser-unprocessed plate, Ex B Case I				
Maximum von Mises stress (MPa)	299	345	383	412
Bending force F_calc_ (N)	1446	1576	1701	1898
Laser-treated plate, Ex B Case II A				
Maximum von Mises stress (MPa)	448	493	524	559
Bending force F_calc_ (N)	1840	2005	2099	2238
Laser-treated plate, Ex B Case II B				
Maximum von Mises stress (MPa)	519	518	555	587
Bending force F_calc_ (N)	1845	2033	2171.9	2295
Laser-treated plate, Ex B Case II C				
Maximum von Mises stress (MPa)	448	502	539	569
Bending force F_calc_ (N)	1853	2036	2159	2282
Laser-treated plate, Ex B Case III A				
Maximum von Mises stress (MPa)	441	484	512	541
Bending force F_calc_ (N)	1591	1742	1852	1954
Laser-treated plate, Ex B Case III B				
Maximum von Mises stress (MPa)	455	491	521	550
Bending force F_calc_ (N)	1623	1776	1909	2030
Laser-treated plate Ex B Case III C				
Maximum von Mises stress (MPa)	443	493	522	556
Bending force F_calc_ (N)	1584	1771	1875	1998
Laser-treated plate, Ex B Case IV A				
Maximum von Mises stress (MPa)	446	481	506	537
Bending force F_calc_ (N)	1584	1724	1820	1947
Laser-treated plate, Ex B Case IV B				
Maximum von Mises stress (MPa)	445	473	582	598
Bending force F_calc_ (N)	1524	1684	1802	1895
Laser-treated plate, Ex B Case IV C				
Maximum von Mises stress (MPa)	444	484	507	535
Bending force F_calc_ (N)	1566	1714	1814	1939

**Table 8:** Tensile modelling results; maximum equivalent stresses and deformations of samples.

Name of the Specimen	Max. Equivalent Stress (MPa)	Strain in the Area L_0_ (mm)	Elongation δ (%)
Ex T Case I (base material)	410.57	5.56	9.3
Ex T Case II A	525.26	3.72	6.2
Ex T Case II B	523.05	3.35	5.6
Ex T Case II C	532.83	3.33	5.6
Ex T Case III A	540.12	4.46	7.4
Ex T Case III B	545.4	4.30	7.2
Ex T Case III C	558.04	5.07	8.4
Ex T Case IV A	541.58	4.74	7.9
Ex T Case IV B	539.14	4.63	7.7
Ex T Case IV C	411.5	5.56	9.3

**Table 9:** Detailed comparison of results of mechanical bend tests and computational modeling Ex B.

Bending Forces (F_exp_, F_calc_) and Relative Differences d_(F,r)_ = (F_exp_ − F_calc_)/F_calc_	Ex B Case I	Ex B Case II A	Ex B Case II B	Ex B Case II C	Ex B Case III A	Ex B Case III B	Ex B Case III C	Ex B Case IV A	Ex B Case IV B	Ex B Case IV C
Deflection, 0.5 mm										
F_exp_ (N)	1325	1760	1696	1745	1497	1569	1495	1517	1444	1493
F_calc_ (N)	1446	1840	1845	1853	1591	1623	1584	1584	1524	1566
d_(F,r)_ (%)	9	4	9	6	6	3	6	4	6	5
Deflection, 1 mm										
F_exp_ (N)	1481	2029	1923	1991	1608	1658	1712	1686	1623	1607
F_calc_ (N)	1576	2005	2033	2036	1742	1776	1771	1724	1684	1714
d_(F,r)_ (%)	6	1	6	2	8	7	3	2	4	7
Deflection, 1.5 mm										
F_exp_ (N)	1530	2145	2088	2042	1659	1744	1800	1798	1708	1723
F_calc_ (N)	1701	2099	2171	2159	1852	1909	1875	1820	1802	1814
d_(F,r)_ (%)	11	2	4	6	12	9	4	1	5	5
Deflection, 2 mm										
F_exp_ (N)	1690	2263	2209	2237	2113	2024	2083	1943	1804	1864
F_calc_ (N)	1898	2238	2295	2282	1954	2030	1998	1947	1895	1939
d_(F,r)_ (%)	12	1	4	2	8	0.3	4	0.2	5	4

**Table 10:** Comparison of all obtained results for mechanical tensile tests and computational modelling.

Name of the Specimen	Calculated Breaking Stress (MPa)	Real Breaking Stress (MPa)	Difference (%)
Ex T Case I (base material)	410.57	400	2.57
Ex T Case II A	525.26	414.5	21.09
Ex T Case II B	523.05	422.3	19.26
Ex T Case II C	532.83	440	17.42
Ex T Case III A	540.12	426.3	21.07
Ex T Case III B	545.4	426	21.89
Ex T Case III C	558.04	433.5	22.32
Ex T Case IV A	541.58	426.5	21.25
Ex T Case IV B	539.14	428	20.61
Ex T Case IV C	411.5	430.5	4.41

## Data Availability

Not applicable.
